# RUNX2 and Cancer

**DOI:** 10.3390/ijms24087001

**Published:** 2023-04-10

**Authors:** Tsung-Chieh Lin

**Affiliations:** 1 Genomic Medicine Core Laboratory, Department of Medical Research and Development, Chang Gung Memorial Hospital, Linkou 333, Taiwan; tclin1980@cgmh.org.tw; Tel.: +886-3-3281200 (ext. 7722); 2 Department of Biomedical Sciences, Chang Gung University, Taoyuan City 333, Taiwan

**Keywords:** *RUNX2*, prognosis, cancer progression

## Abstract

Runt-related transcription factor 2 (RUNX2) is critical for the modulation of chondrocyte osteoblast differentiation and hypertrophy. Recently discovered RUNX2 somatic mutations, expressional signatures of *RUNX2* in normal tissues and tumors, and the prognostic and clinical significance of RUNX2 in many types of cancer have attracted attention and led RUNX2 to be considered a biomarker for cancer. Many discoveries have illustrated the indirect and direct biological functions of RUNX2 in orchestrating cancer stemness, cancer metastasis, angiogenesis, proliferation, and chemoresistance to anticancer compounds, warranting further exploration of the associated mechanisms to support the development of a novel therapeutic strategy. In this review, we focus mainly on critical and recent research developments, including RUNX2’s oncogenic activities, by summarizing and integrating the findings on somatic mutations of *RUNX2*, transcriptomic studies, clinical information, and discoveries about how the RUNX2-induced signaling pathway modulates malignant progression in cancer. We also comprehensively discuss *RUNX2* RNA expression in a pancancer panel and in specific normal cell types at the single-cell level to indicate the potential cell types and sites for tumorigenesis. We expect this review to shed light on the recent mechanistical findings and modulatory role of RUNX2 in cancer progression and provide biological information that can guide new research in this field.

## 1. Introduction

**Figure 1:**
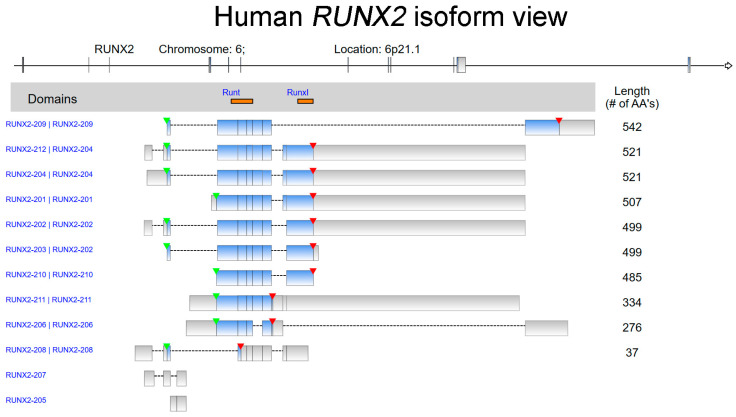
The isoform view of human *RUNX2*. The red and green arrowheads show the positions of the stop codon and transcription start site, respectively. In addition, the matched protein domains in each isoform are marked in orange. Runt: Runt domain. RunxI: Runx inhibition domain. The information is based on the data adapted with permission from Ingenuity Pathway Analysis. Copyright Year 2023, QIAGEN.

In 1993, a gene family of DNA-binding transcriptional regulatory proteins was identified from *Drosophila*, mouse, and human. These genes shared a highly conserved, 128-amino-acid region characterized as a DNA-binding domain, Runt [1]. Three members, *RUNX1/Cbfa1/Pebp2αA*, *RUNX2/Cbfa2/Pebp2αB*, and *RUNX3/Cbfa3/Pebp2αC*, in this Runt-domain family were further reported in humans [2,3,4]. The human *RUNX2* (Runt-related transcription factor 2) gene was isolated from a B-cell-derived cDNA library [2]. The *RUNX2* gene is located at 6p21.1 in humans [5] and encodes various isoforms with a total of 12 transcript variants (Figure 1). RUNX2 is involved in osteogenesis and the maturation of chondrocytes via the modulation of transcriptional activation and multiple signaling pathways [6,7,8,9]. RUNX2 is a well-known master regulator of osteoblast and chondrocyte differentiation, but new findings have demonstrated its participation in cancer progression and tumorigenesis. Accumulated experimental data have revealed the functions of the RUNX2-mediated downstream axis in modulating angiogenesis, cancer metastasis, proliferation, cancer stemness, and drug resistance leading to cancer progression. With this review article, we aim to summarize the up-to-date information about RUNX2-related biological functions in pancancer. The *RUNX2* RNA level in normal cells and tissues is first demonstrated at the single-cell level. We integrate and summarize the current research findings on this topic, focusing on somatic mutations of the *RUNX2* gene and evidence indicating *RUNX2* expression levels in different tumor types as well as the RUNX2-dependent biological effects in orchestrating cancer progression. In addition, the prognostic and clinical significance of *RUNX2* is presented in a pancancer panel.

## 2. RUNX2 and Cancer Proliferation

In thyroid cancer, the biological significance of RUNX2 in follicular thyroid cancer ML-1 cells was investigated. Knockdown of *RUNX2* by siRNA revealed a decrease in cancer cell proliferation accompanied by an increase in store-operated calcium entry (SOCE) [10]. In ccRCC, RUNX2 triggered cancer cell proliferation via SCD1-dependent Wnt/β-catenin pathway activation. RUNX2 downregulation inhibited cancer cell proliferation [11]. In another study of ccRCC, the biological function of RUNX2 was to increase focus formation, Ki67-positive staining, and tumor volume in a xenograft model. The effect was mediated by the repression of a tumor suppressor, nucleolar, and coiled-body phosphoprotein 1 (NOLC1) [12]. When RUNX2-orchestracted breast cancer growth was studied, RUNX2 silencing in a breast cancer cell line inhibited cancer proliferation in a plate cloning assay and in a subcutaneous neoplasia model of BALB/c nude mice [13]. Consistent with that report, the depletion of RUNX2 by siRNA inhibited proliferation, as determined by a 5-ethynyl-20-deoxyuridine (EdU) assay, in MDA-MB-231 and SUM159 breast cancer cells. Tumorigenicity was also blocked. Tumor-initiating capacity was lower in the group with MDA-MB-231-shRUNX2 cells injected into NOD/SCID mice via the mammary gland fat pads. The effect was mediated by the recruitment of the NuRD(MTA1)/CRL4B complex by RUNX2 to form a transcriptional repressive complex [14]. In OSCC, RUNX2 gene silencing abolished the malignant progression, and a reduction in EdU positivity in both CAL-27 and TSCCA cells was observed [15]. In pancreatic adenocarcinoma, PI3K/AKT and MAPK signaling might be modulated by RUNX2 to augment cell growth [16]. In colorectal cancer, the cell proliferation markers Ki-67 and PCNA were downregulated upon RUNX2 silencing, and RUNX2 was required for CBFβ-elicited cell proliferation. Mechanistically, RUNX2 and CBFβ form a transcriptional complex that binds to promoters and contributes to the upregulation of downstream genes, including OPN, FAM129A, and UPP1, in colorectal cancer HCT116 cells [17]. The association of RUNX2 with cancer-associated fibroblast infiltration as well as epithelial–mesenchymal transition was reported in a bladder urothelial cancer study. Knockdown of RUNX2 diminished the proliferation rate of cancer cells [18]. RUNX2 is involved in the long noncoding RNA HLA complex group 18 (LncRNA HCG18)-elicited tumorigenic phenotype in osteosarcoma. LncRNA HCG18 silencing led to a decrease in cancer cell proliferation, which effect was abolished by RUNX2 overexpression [19]. The regulatory link between miRNA-218 and RUNX2 was noted in osteosarcoma U2OS cell proliferation [20]. RUNX2 upregulation was characterized as a downstream event of circRANBP17 in nasopharyngeal carcinoma. Overexpression of RUNX2 in a rescue assay facilitated cancer cell proliferation, as judged by increased EdU-positive rates [21]. In lung cancer, RUNX2 had an antiapoptotic effect, and a shRNA-mediated loss-of-function experiment resulted in increased dead cancer cells determined by positive annexin/propidium iodide (PI) in flow cytometry. RUNX2′s biological impact was shown by direct binding to the promoter region of the antiapoptotic gene *BCL2* in a chromatin immunoprecipitation (ChIP) assay, resulting in its transcriptional activation, and by the indirect modulation of *BCL-XL* and *MCL1* [22].

## 3. RUNX2 and Angiogenesis

In the examination of 89 human hepatocellular carcinoma samples, RUNX2 expression appeared to correlate with vasculogenic mimicry (VM), the mimicry of endothelial cells by cancer cells to form the microvascular structure in aggressive tumors. Overexpression of RUNX2 further resulted in VM formation of HepG2 cells [23]. The Runt domain of RUNX2 is critical for its function in stimulating angiogenesis. 3G8 melanoma cells with the Runt domain knocked out by the CRISPR/Cas9 system decreased VEGA and abolished the tubular-like structure formation ability in HUVECs as well as the expression of the neoangiogenetic markers CD105 and CD31 in a coculture system [24]. RUNX2 overexpression in prostate cancer LNCaP cells increased tumor angiogenesis and oxygenation in vivo in a xenograft model [25]. In a study of multiple myeloma, DNA binding activity and induction of osteopontin expression by RUNX2 were reported, which contributed to the proangiogenic effect of RPMI-8226 cells in vitro [26]. In neuroblastoma cells, YAP-RUNX2-SRSF1-VEGFA signaling was altered by the stiffness of the extracellular matrix, and this axis had proangiogenic effects in the form of increased tube formation in vitro, as shown by 3D Col-Gel implantation in nude mice in vivo. RUNX2 was required to modulate SRSF1 expression [27]. In a study exploring the impact of emodin treatment on alleviating breast cancer cell angiogenesis, the reduced phosphorylation activation, but not expression, of RUNX2 as well as the disrupted DNA binding activity measured by ELISA were detected in MDA-MB-231 and endothelial cells upon emodin stimulation, respectively [28]. In a mechanical investigation of codonolactone-inhibited cancer angiogenesis, a BMP-RUNX2-MMPs/VEGF axis was proposed as a critical route upon angiogenesis of endothelial cells [29]. In endothelial cells, RUNX2′s DNA binding activity and the angiogenic phenotype were regulated by hyperglycemia, that is, the glucose-mediated intracellular pathway and redox status, suggesting the potential role of RUNX2 in orchestrating tumor-associated angiogenesis [30]. In endothelial cells, RUNX2 phosphorylation at the C-terminal domain (Ser451) was found to be critical for its DNA binding activity, monolayer wound healing, and in vitro tube formation, and these functions were blocked by the S451A mutation [31].

## 4. RUNX2 and Cancer Metastasis

In renal cell carcinoma, RUNX2 appeared to promote cancer cell invasion through the calpain2–fibronectin axis, and the RUNX2-overexpression-mediated effect was attenuated by the calpain inhibitor calpeptin or calpain2 small interfering RNA (siRNA) in A498 cells [32]. The role of RUNX2 in the clear cell subtype of RCC (ccRCC) cell migration was addressed. RUNX2 overexpression led to an increase in cell migration ability, and this elevated migration was partially reduced by the downregulation of SCD1 [11]. Activation of Zic2/Runx2/NOLC1 signaling promoted ccRCC cell migration and lung metastasis in vivo [12]. The regulation of RUNX2 toward the extracellular matrix component collagen type I alpha 1 (COL1A1) was revealed in a gastric cancer study. RUNX2 overexpression induced COL1A1 expression and promoted cancer cell migration and invasion in vitro and in an animal model of metastasis via COL1A1 [33]. In thyroid cancer, a reduction in invasion activity was detected after silencing RUNX2 by siRNA in ML-1 cells [10]. Stable knockdown of RUNX2 in a triple-negative breast cancer cell line (MDA-MB-231) resistant to epirubicin abolished the cell invasion and migration activity, determined by Transwell assay. In addition, RUNX2 overexpression led to the upregulation of MMP1, which might degrade the extracellular matrix in the tumor microenvironment. Direct binding of RUNX2 to the *MMP1* promoter region was detected, suggesting the potential significance of the RUNX2-MMP1 axis in cancer progression [13]. Another breast cancer study indicated that RUNX2 could induce the invasion and further drive the adhesion and attraction of cancer cells to bone via the inhibition of SOD2 and PPARα expression [14]. An analysis of chromatin accessibility indicated RUNX2 as a master transcription factor in a distinct cell population with high Wnt signaling activity. RUNX2 was further found to elicit the metastasis of colon cancer cells in vivo [34]. The invasion and migration capability of colorectal cancer cells were positively regulated by RUNX2 via a RUNX2-BRG1 complex and the CD44 signaling pathway [35]. In OSCC (TSCCA and CAL-27 cells), RUNX2 silencing abolished the malignancy by inhibiting the cells’ invasion ability [15]. In pancreatic adenocarcinoma, knockdown of RUNX2 expression by specific shRNA caused a decrease in ASPC-1 cell migration, accompanied by phosphorylation activation of the MAPK and PI3K/AKT axes [16]. In osteosarcoma, tumor cell invasion and migration were triggered by lncRNA HCG18 via the repression of miR-34a, a negative regulator of RUNX2, which increased RUNX2 levels [19]. A similar function of RUNX2 was reported elsewhere: overexpression of RUNX2 in U2OS osteosarcoma cells reversed the effect mediated by miRNA-218, a direct interactive target of Runx2, and promoted cancer cell migration and invasion [20]. In nasopharyngeal carcinoma, RUNX2 overexpression abolished the effect of circRANBP17-dependent suppression on cancer cell invasion [21]. CBFβ was characterized by its biological function in promoting cell migration and invasion in colorectal cancer cells, and the modulation depended on RUNX2 [17]. An indirect inhibitory role of RUNX2 was also reported. RUNX2 expression appeared to be repressed by caveolin-1, a major structural protein of caveolae; the RUNX2-induced transcription of miR24 was attenuated during caveolin-1-mediated cell invasion in hepatocellular carcinoma [36].

## 5. *RUNX2* and Drug Resistance

*RUNX2*′s functional significance in drug resistance was reported in a triple-negative breast cancer study. Relative RUNX2 protein expression was higher in the MDA-MB-231-Re (epirubicin-resistant) cell line than in the MDA-MB-231 parental cells. RUNX2 knockdown in MDA-MB-231-Re cells further weakened resistance to epirubicin treatment in a CCK-8 cell viability assay [13]. In a study of osteosarcoma, RUNX2 was knocked down in MG63 and U2OS cells, which sensitized osteosarcoma cells to the chemotherapy treatment of cisplatin [37]. miR-218-RUNX2 signaling was involved in modulating the efficacy of chemotherapy in non-small-cell lung cancer. RUNX2 silencing in A549 cells increased their sensitivity to cisplatin in vitro [38]. In human osteosarcoma-derived U2OS cells, adriamycin-mediated cell death depended on various p53/TAp73 target gene products, and TAp73 was repressed by RUNX2 overexpression. RUNX2 appeared to form a complex with TAp73 and impair its transcriptional activity [39]. Anoikis-resistant osteosarcoma cells were resistant to standard chemotherapy with doxorubicin and cisplatin. A comprehensive screening of altered gene expression in the cells identified the upregulation of RUNX2, indicating its potential role in orchestrating drug resistance [40]. In prostate cancer, the increased expression of RUNX2 under nitric oxide conditions conferred resistance to docetaxel in LNCaP cells, and activation of the ERK-PI3K-AP1-RUNX2 axis was indicated [25]. In contrast to previous findings, a role for RUNX2 in alleviating drug resistance was reported: in multiple myeloma, a mouse model with specific RUNX2 deficiency in osteoblasts (RUNX2^−/−^) rendered multiple myeloma cells more resistant to bortezomib via thrombospondin-1-mediated TGFβ1 activation, whereas the malignancy and tumor burden were reversed by treatment with the antagonist SRI31277 [41].

## 6. RUNX2, Transdifferentiation, and Cancer Stemness

RUNX2 modulated cancer stemness in a breast cancer study: RUNX2 overexpression in MDA-MD-231 cells led to an increase in sphere volume in a spheroid-forming assay [14]. Breast cancer stem cells characterized as CD44^+^/CD24^−/low^ were regulated by RUNX2. RUNX2 overexpression in MCF-7 cells led to an increase in this population in a flow cytometry experiment as well as the induction of sphere formation. RUNX2-mediated malignancy was shown by the increased tumor growth in nude mice in a xenograft model [42]. In a colon cancer study, RUNX2 was found to trigger epithelial–mesenchymal transition (EMT) in vitro through orchestration of the chromatin landscape and the expression of EMT-related genes [34]. The results from an additional colorectal cancer study indicated the ability of RUNX2 to induce EMT and sphere formation in cancer. RUNX2 interacted with BRG1 to form a compact complex contributing to promoter recruitment and transcriptional activation of CD44, a biomarker of cancer stem cells [35]. A pro-EMT role for the RUNX2/STK32A/NF-κB p65 axis was uncovered in non-small-cell lung cancer (NSCLC). RUNX2 appeared to combine with STK32A to promote its expression, leading to NF-κB p65 phosphorylation [43].

## 7. *RUNX2* Somatic Mutation and Cancer

**Figure 2:**
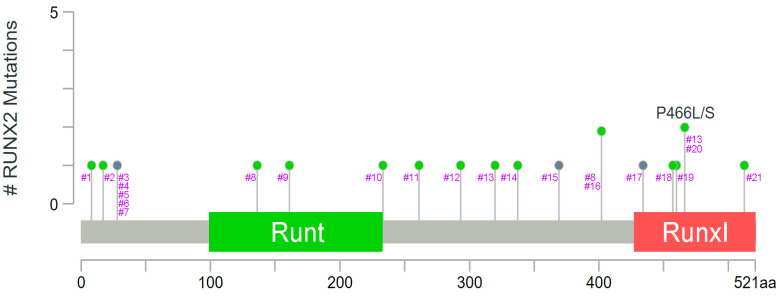
A pancancer study addressing whole genome data reveals the sites and types of *RUNX2* mutations. Gray indicates the truncating mutations (putative driver) including nonsense, nonstop, frameshift deletion, and frameshift insertion. Light green indicates the missense mutations. Runt: Runt domain. RunxI: Runx inhibition domain. Data were adapted with permission from cBioPortal (https://docs.cbioportal.org/user-guide/faq/#can-i-use-figures-from-the-cbioportal-in-my-publications-or-presentations) accessed on: 21 February 2023.

Genetic variants of the *RUNX2* gene in patients with cancer have been comprehensively addressed reported. A pancancer analysis that integrated 2658 whole-cancer genome data as well as the matched normal tissues from 38 tumor types has been published [44]. *RUNX2* mutations in various cancer types were collected and listed based on the data from the database cBioPortal (https://www.cbioportal.org/) [45,46] (Figure 2 and Table 1, accessed on: 21 February 2023). *FOS-RUNX2* gene fusion was reported in osteoblastoma samples. The C-terminal part of FOS, involved in the FOS degradation process, was removed in the chimeric protein. The fusion event also led to the deletion of the 3′-untranslated region of FOS mRNA, which is required for its interaction with miRNA [47]. In an investigation by a capture-based next-generation sequencing (NGS) platform, copy number changes in 111 osteosarcoma patients were analyzed. *RUNX2* at 6p21.1 was found within the amplified locus, and amplification was confirmed by fluorescence in situ hybridization [48]. In another osteosarcoma study, amplification of *RUNX2* was found in both the primary tumor and the metastatic tumor [49]. *RUNX2* gene amplification was also discovered in 16 of 21 metastatic conjunctival melanomas via DNA analysis by multiplex ligation-dependent probe amplification assays [50].

## 8. *RUNX2* Distribution and Expression in Normal Cell Types

**Figure 3:**
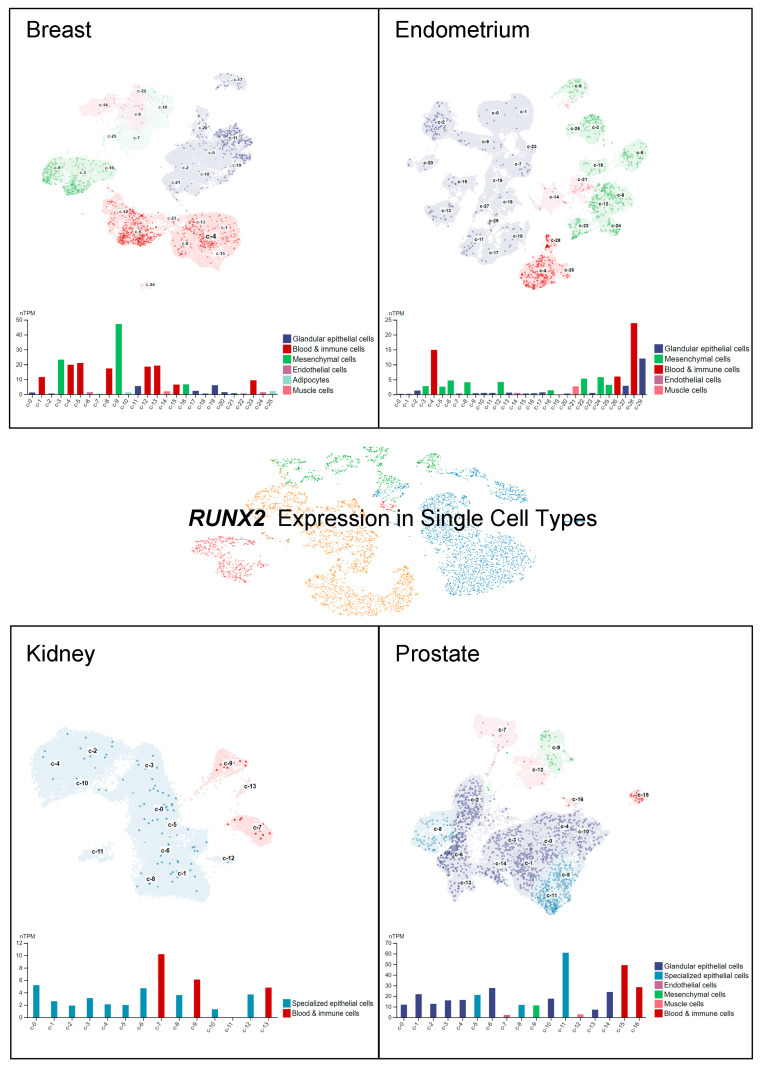
Human *RUNX2* RNA distribution in single cells across different cell types. The *RUNX2* level was measured by scRNA-seq in different tissues. The RNA expression levels are visualized at the single-cell level by UMAP plot. Data were adapted with permission from HPA (https://www.proteinatlas.org/about/licence#citation_guidelines_for_the_human_protein_atlas, accessed on: 21 February 2023).

**Figure 4:**
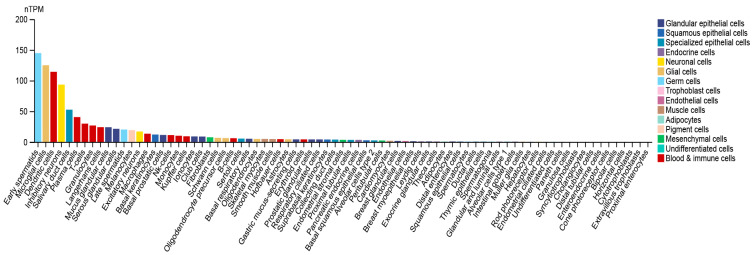
Relative *RUNX2* expression across pan-normal cell types. *RUNX2* RNA levels were measured by scRNA-seq in 192 specific cell type clusters. nTPM: TPM values of all samples were normalized separately using the trimmed mean of M values (TMM) to allow for between-sample comparisons and normalized transcript expression values. Data were adapted with permission from HPA (https://www.proteinatlas.org/about/licence#citation_guidelines_for_the_human_protein_atlas, accessed on: 21 February 2023).

*RUNX2* RNA expression level has been explored by single-cell RNA sequencing (scRNA-seq) to dissect its distribution among specific cell types in a given tissue [51,52,53,54]. Importantly, single-cell RNA expression might shed light on the further investigation of tumorigenesis and identification of specific biomarkers in cancer [55]. A recently released cell type atlas illustrated the scRNA-seq data of specific gene expression in 192 specialized clusters/cell types (Human Protein Atlas, https://www.proteinatlas.org/, accessed on: 21 February 2023) [56]. *RUNX2* expression levels in normal organs, namely, the breast, endometrium, kidney, and prostate, are indicated at the single-cell level in Figure 3. A relatively higher *RUNX2* expression has been detected in mesenchymal cells and blood and immune cells in the breast. Blood and immune cells and glandular epithelial cells in the endometrium have been found to display significant *RUNX2* expression. In addition, *RUNX2* expression in the kidney has been specifically observed in specialized epithelial cells and blood and immune cells, but not in other cell types. In prostate tissues, *RUNX2* RNA expression has been found in specialized epithelial cells, blood and immune cells, and glandular epithelial cells. These research findings suggest the potential sites of RUNX2-mediated downstream events that may occur and lead to tumorigenesis. Furthermore, we provide an overall demonstration of *RUNX2* RNA distribution across all normal cell types. The top five specific cell types with high *RUNX2* levels are identified as early spermatids, microglial cells, dendritic cells, inhibitory neurons, and salivary duct cells (Figure 4).

## 9. RUNX2 Expression in Cancers

**Figure 5:**
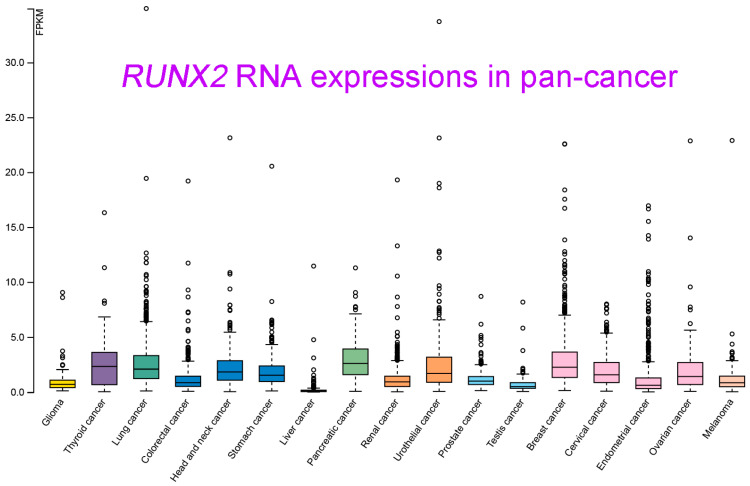
*RUNX2* RNA-seq data in 17 cancer types (TCGA) were re-analyzed. These transcript expression data were obtained by RNA-Seq analyses based on the data retrieved from the TCGA database and were normalized and used to assess relative RUNX2 expression in various types of cancers. Data are shown as the median number of fragments per kilobase per million (FPKM). Normal distribution in the dataset is represented by the box plots, and the points represent the data of outliers if the expression levels are below or above 1.5 times the interquartile range. Data were adapted with permission from HPA (https://www.proteinatlas.org/about/licence#citation_guidelines_for_the_human_protein_atlas, accessed on: 21 February 2023).

RUNX2 RNA and protein expression levels in various types of cancer were measured. Relatively high RUNX2 levels were detected by IHC staining in tissues of renal cell carcinoma compared with nontumor tissue, whose regulatory mechanism required Zic family member 2 (Zic2) in 786-O and ACHN cells [12]. RUNX2 was shown to be an interactive target of miR-23a-3p in CAL-27 cells and TSCCA cells, and oral squamous cell carcinoma (OSCC) overexpressing miR-23a-3p mimics decreased the RUNX2 level [15]. RUNX2 was significantly decreased by transfection of a miRNA-218 mimic, and RUNX2 expression was obviously increased by treatment with a miRNA-218 inhibitor in osteosarcoma U2OS cells [20]. In oral cancer (both HSC-3 and Ca9-22 cells), RUNX2 expression was positively regulated by MRE11, the nuclease component of the RAD50/MRE11/NBS1 DNA repair complex [57]. In a colorectal cancer study that enrolled 75 cancer patients, cancer tissues displayed high RUNX2 levels compared with normal adjacent tissues. Consistent results with these were obtained by Western blot analysis of 10 paired cancer and normal tissues [17]. RUNX2 protein was detected in cervical cancer tissues, and RUNX2 expression declined upon overexpression of miR-218-5p in C-33A and CaSki cells [58]. RUNX2 protein was elevated in human thyroid cancer cell lines and cancer tissues compared with primary cell lines and normal thyroid tissues [10]. RUNX2 was overexpressed in lung adenocarcinoma in a large study that included 2418 tumor and 1574 nontumor lung samples [59]. In gastric cancer, RUNX2 expression levels were analyzed by immunohistochemical staining of 60 cancer tissues and by consulting the Gene Expression Profiling Interactive Analysis (GEPIA) database, which demonstrated the high expression of RUNX2 at both the gene and protein levels in gastric cancer [33]. In oral squamous cell carcinoma (OSCC), RUNX2 RNA levels were found to be statistically higher in tumor tissues than in normal tissues by qRT-PCR analysis of 40 pathological specimens. A similar result was observed in a comparison between squamous cell carcinoma cells (TCA8113, CAL-27, SCC-9, and TSCCA) and normal oral keratinocytes (NHOK) [15]. Nickel (Ni) compounds are classified as Group 1 carcinogens, including to the lungs. RUNX2 expression appeared to be increased upon Ni-initiated BEAS-2B transformation, suggesting a potential role in lung tumorigenesis [60]. RUNX2 expression could also be orchestrated by circular RNA (circRNA)-mediated signaling. In nasopharyngeal carcinoma, circRANBP17 promoted RUNX2 expression by sponging miR-635 [21]. RUNX2 was overexpressed in tissue samples of bladder urothelial cancer, and immunohistochemistry further demonstrated the positive correlation of high RUNX2 levels with cancer-associated fibroblast biomarkers [18]. The data of integrating the transcriptomic studies in various cancer types and the matched clinical information were announced and released (University of California, Santa Cruz, *n* = 12,839) [61]. As seen in Figure 5, *RUNX2* was shown to be highly upregulated in pancreatic cancer, breast cancer, lung cancer, thyroid cancer, and head and neck cancer. In contrast, lower *RUNX2* levels were detected in liver cancer and testis cancer.

## 10. Correlation with Clinical Outcome

RUNX2 appears to be a prognostic biomarker in many cancer types. In oral cancer patients, a high RUNX2 level was correlated with lymph node metastasis [57]. Tumor budding has been characterized as a microscopic-finding-based dedifferentiation at the invasive margin in colon cancer. RUNX2 was identified as a constituent of the molecular budding gene signature and contributed to unfavorable relapse-free survival rates in a cohort study of 85 patients with stage II/III disease [62]. In an exploration of clinical data in colon cancer, RUNX2 was expressed higher in cancer patients with metastasis and shorter survival [34]. In a clinical study of gastric cancer, patients with positive RUNX2 expression had unfavorable survival, clinical stage, and associated lymph node metastasis [33]. RUNX2 expression was measured by immunohistochemistry and analyzed for correlations with clinical data in 105 osteosarcoma patients, and it appeared to be an independent predictor of metastasis-free survival and overall survival in a multivariate survival analysis. In addition, RUNX2 and osteopontin expression were strongly correlated at the protein level [63]. In lung adenocarcinoma, the expression of RUNX2 correlated with a poor hazard ratio, suggesting that RUNX2 plays a clinical role as an independent risk factor for poor survival in lung cancer [59]. A similar result demonstrated the positive correlation of elevated RUNX2 with poor overall survival of non-small-cell lung cancer patients [64]. RUNX2 expression was associated with adverse overall survival in a study of 301 renal cell carcinoma patients. In addition, correlations with poor grade and stage were revealed by an analysis of the TCGA database [12]. In hepatocellular carcinoma, the data from clinicopathological analysis of 89 samples indicated the correlation of RUNX2 expression with metastasis rate and shorter survival period [23]. An immunohistochemistry-based study of breast cancer tissue samples obtained from 75 patients showed that a high RUNX2 level was significantly associated with poor prognosis, Ki-67 expression, and lymphatic metastasis [65]. A comprehensive pancancer study integrating cancer patients’ clinical data with RNA expression profiles has been completed and released from the Human Protein Atlas (HPA) [56,66,67,68,69] and Kaplan–Meier plotter [70] databases. The prognostic data of *RUNX2* in different cancer types are listed in Table 2 (data were adapted with permission from HPA: https://www.proteinatlas.org/about/licence#citation_guidelines_for_the_human_protein_atlas, accessed on 21 February 2023). *RUNX2* appears to be an inferior prognostic biomarker in cohorts of patients with glioma, colorectal cancer, stomach cancer, pancreatic cancer, renal cancer, urothelial cancer, lung cancer, and cervical cancer. On the other hand, in patients diagnosed with breast and ovarian cancer determined by array, high *RUNX2* expression levels are correlated with better clinical outcomes.

## 11. Summary

RUNX2 protein has a highly conserved DNA-binding domain, known as a Runt domain. The Runt domain appears to form heterodimerizes with a common non-DNA-binding core binding factor beta (CBF-β) subunit. This interaction could result in a structural change to enhance the binding of RUNX2 to the consensus DNA-binding motif [71]. In addition, relative regulations by its interactors were reported. Zic2 was required for RUNX2′s high expression in renal cell carcinoma [12]. miR-23a-3p was an interactive target of *RUNX2* and decreased its level in OSCC [15]. RUNX2 expression was found to be increased by MRE11 in oral cancer [57]. RUNX2 expression could also be modulated by circRNA. In nasopharyngeal carcinoma, circRANBP17 was found to increase RUNX2 levels by sponging miR-635 [21]. In addition, RUNX2 could be modulated by multiple post-translational modifications including phosphorylation by Erk [72,73], acetylation by histone acetyltransferases [74], and prolyl isomerization by Pin1 [75].

**Figure 6:**
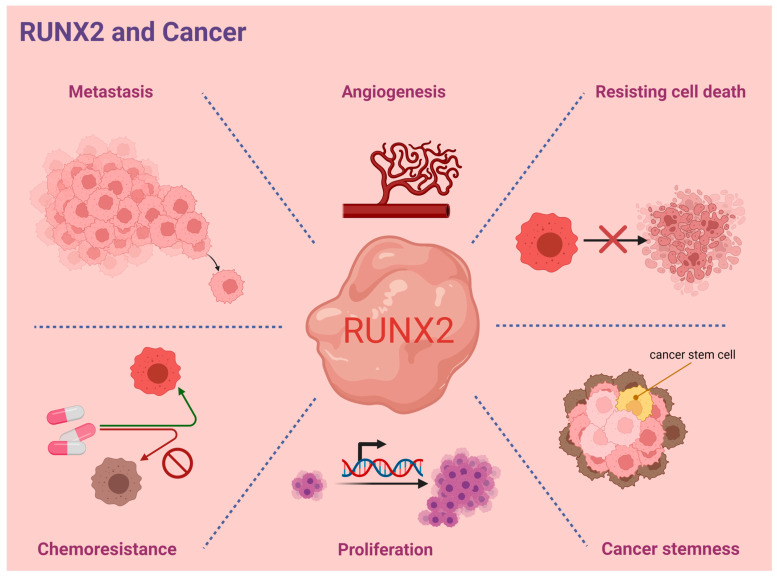
Representative scheme of RUNX2′s modulations to hallmarks of cancer.

Concurrent and emerging studies indicate the critical role of RUNX2 in orchestrating cancer progression in various tumor types. RUNX2-guided signaling axes were found to participate in the modulation of several key processes of cancer progression including transdifferentiation and cancer stemness, angiogenesis, cancer cell metastasis, proliferation, and drug resistance. These experimental observations suggest the multifunctional role of RUNX2 in cancer progression, which is summarized in Figure 6. However, a potentially protective role of RUNX2 in cancer has also been reported. In caveolin-1-dependent hepatocellular carcinoma invasion, RUNX2 expression appeared to be suppressed by caveolin-1 along with the repression of RUNX2-induced miR24 transcription [36]. Importantly, RUNX2-associated discrepancies in biological effects might also be determined by RUNX2’s interactive cofactors, which remains to be explored.

## Figures and Tables

**Table 1:** *RUNX2* mutations in a pancancer study of whole genomes.

Sample ID	Cancer Type	Protein Change	Mutation Type	Variant Type	Copy Number	Mutations in Sample
#1	Uterine Endometrioid Carcinoma	S8N	Missense	SNP	Diploid	568
#2	Esophagogastric Cancer	F17V	Missense	SNP	Gain	141
#3	Prostate Cancer	S31Lfs*130	FS ins	INS	Gain	41
#4	Prostate Cancer	S31Lfs*130	FS ins	INS	Gain	48
#5	Prostate Cancer	S31Lfs*130	FS ins	INS	Gain	55
#6	Prostate Cancer	S31Lfs*130	FS ins	INS	Gain	54
#7	Prostate Cancer	S31Lfs*130	FS ins	INS	Gain	50
#8	Ovarian Cancer	L136V	Missense	SNP	Gain	63
#9	Colorectal Cancer	D161N	Missense	SNP	Diploid	4888
#10	Ovarian Cancer	K233E	Missense	SNP	Amp	102
#11	Uterine Endometrioid Carcinoma	V261A	Missense	SNP	Diploid	1321
#12	Hepatobiliary Cancer	S293P	Missense	SNP	Diploid	68
#13	Melanoma	P320L	Missense	SNP	Amp	1401
#14	Lung Cancer	R337M	Missense	SNP	Gain	80
#15	Embryonal Tumor	S371Ffs*14	FS ins	INS	Diploid	17
#16	Glioma	P402L	Missense	SNP	Diploid	77
#8	Ovarian Cancer	P402T	Missense	SNP	Gain	63
#17	Breast Cancer	C434*	Nonsense	SNP	Diploid	82
#18	Endometrial Cancer	E458K	Missense	SNP	Gain	256
#19	Pancreatic Cancer	G459V	Missense	SNP	Diploid	62
#20	Melanoma	P466L	Missense	SNP	Diploid	950
#13	Melanoma	P466S	Missense	SNP	Amp	1401
#21	Pancreatic Cancer	G512D	Missense	SNP	Diploid	55

FS: frameshift mutation; INS: insertion; *: nonsense mutation.

**Table 2:** Correlation of *RUNX2* with cancer patient survival.

Symbol	Cancer Type	Prognosis	Endpoint	*p* Value	Case	Dataset	Method	Probe ID
*RUNX2*	Glioma	Poor	Overall survival	0.02	153	TCGA	RNA Seq	
*RUNX2*	Thyroid Cancer	-	Overall survival	N.S.	501	TCGA	RNA Seq	
*RUNX2*	Lung Cancer	-	Overall survival	N.S.	994	TCGA	RNA Seq	
*RUNX2*	Colorectal Cancer	Poor	Overall survival	0.04	597	TCGA	RNA Seq	
*RUNX2*	Head and Neck Cancer	-	Overall survival	N.S.	499	TCGA	RNA Seq	
*RUNX2*	Stomach Cancer	Poor	Overall survival	<0.001	354	TCGA	RNA Seq	
*RUNX2*	Liver Cancer	-	Overall survival	N.S.	365	TCGA	RNA Seq	
*RUNX2*	Pancreatic Cancer	Poor	Overall survival	0.037	176	TCGA	RNA Seq	
*RUNX2*	Renal Cancer	Poor	Overall survival	<0.001	877	TCGA	RNA Seq	
*RUNX2*	Urothelial Cancer	Poor	Overall survival	<0.001	406	TCGA	RNA Seq	
*RUNX2*	Prostate Cancer	-	Overall survival	N.S.	494	TCGA	RNA Seq	
*RUNX2*	Testis Cancer	-	Overall survival	N.S.	134	TCGA	RNA Seq	
*RUNX2*	Breast Cancer	-	Overall survival	N.S.	1075	TCGA	RNA Seq	
*RUNX2*	Cervical Cancer	Poor	Overall survival	0.0089	291	TCGA	RNA Seq	
*RUNX2*	Endometrial Cancer	-	Overall survival	N.S.	541	TCGA	RNA Seq	
*RUNX2*	Ovarian Cancer	-	Overall survival	N.S.	373	TCGA	RNA Seq	
*RUNX2*	Melanoma	-	Overall survival	N.S.	102	TCGA	RNA Seq	
*RUNX2*	Breast Cancer	Good	Relapse-free survival	<0.001	4929	E-MTAB-365, E-TABM-43, GSE: 11,121, 12,093,	Array	216994_s_at
						12,276, 1456, 16,391, 16,446, 16,716, 17,705, 17,907,		
						18,728, 19,615, 20,194, 20,271, 2034, 20,685, 20,711,		
						21,653, 22,093, 25,066, 2603, 26,971, 29,044, 2990,		
						31,448, 31,519, 32,646, 3494, 36,771, 37,946, 41,998,		
						42,568, 43,358, 43,365, 45,255, 4611, 46,184, 48,390,		
						50,948, 5327, 58,812, 61,304, 65,194, 6532, 69,031,		
						7390, 76,275, 78,958, 9195		
*RUNX2*	Ovarian Cancer	Good	Progression-free survival	0.0037	1435	GSE: 14,764, 15,622, 18,520, 19,829, 23,554, 26,193,	Array	216994_s_at
						26,712, 27,651, 30,161, 3149, 51,373, 63,885, 65,986,	RNA Seq	
						9891, TCGA (N = 565)		
*RUNX2*	Lung Cancer	Poor	Postprogression survival	<0.001	1925	CAARRAY, GSE: 14,814, 19,188, 29,013, 30,219,	Array	216994_s_at
						31,210, 3141, 31,908, 37,745, 43,580, 4573, 50,081,	RNA Seq	
						8894, TCGA (N = 133)		
*RUNX2*	Gastric Cancer	Poor	Postprogression survival	<0.001	875	GSE: 14,210, 15,459, 22,377, 29,272, 51,105, 62,254	Array	216994_s_at

Survival data were collected from the Human Protein Atlas, TCGA, and Kaplan–Meier plotter databases. N.S.: no significance.

## Data Availability

All data are contained within the article.
